# Differences in perceptual representations in multilinguals’ first, second, and third language

**DOI:** 10.3389/fnhum.2024.1408411

**Published:** 2024-07-01

**Authors:** Donggui Chen, Jingan Su, Ruiming Wang

**Affiliations:** ^1^School of Foreign Languages, Guangdong Polytechnic Normal University, Guangzhou, China; ^2^Philosophy and Social Science Laboratory of Reading and Development in Children and Adolescents, Ministry of Education, & Center for Studies of Psychological Application, School of Psychology, South China Normal University, Guangzhou, China; ^3^Guangzhou No.18 Middle School, Guangzhou, China

**Keywords:** perceptual representation, embodied cognition, multilinguals, bilinguals, language comprehension

## Abstract

Two experiments were conducted to investigate the differences in perceptual representations among multilingual individuals. In Experiment 1, the immediate sentence-picture verification paradigm was used to investigate perceptual representations in the working memory stage. The results suggest a match effect within the first language (Cantonese), but not within the second language (Mandarin) or the third language (English), showing perceptual representations only in first language comprehension. In Experiment 2, the delayed sentence-picture verification paradigm was used to investigate perceptual representations in long-term memory. Similarly, the results suggest a match effect within the first language (Mandarin), but not within the second language (English). The findings of both experiments suggest that the first language was perceptually represented, regardless of whether it was Cantonese or Mandarin, regardless of the processing in working memory or long-term memory. No evidence was found for perceptual representations in the later-learned languages, regardless of high or low proficiency. Our study has implications for theories of language comprehension and embodied cognition.

## 1 Introduction

Multilingualism is a phenomenon that is observed across the globe. It is estimated that more than half of the world’s population engages in daily communication in a second language (Eberhard et al., 2019). In what manner do different languages coexist within one mind of an individual with multilingual ability? The relationship between language, concepts, and the real world is a topic of significant interest in the field of linguistics. Does the mental representation of one’s mother tongue differ from that of a language acquired later in life? The nature of conceptual representation remains inadequately understood in first language, and even more so in second language (Vukovic and Shtyrov, 2014). One of the most significant debates in the field of cognitive science concerns the nature of the mental representation of concepts. The debate centers on the question of whether the conceptual representation is symbolic or perceptual (Kaup et al., 2024).

The classical view of the representation of concepts is that they are represented in the form of propositions (Fodor, 1975; Pylyshyn, 1985). In contemporary linguistics, language is conceptualized as a symbolic system in which the relationship between the signifier (e.g., a sound, a word or a phrase) and the signified (e.g., objects in the world) is arbitrary. The lower-level processes of perception and action play no role in the formation of cognition (Ojemann, 1991). Cognition is constructed from abstract, amodal representations, which are linked to their referents through formal rules (Newell and Simon, 1972; Collins and Loftus, 1975). From this perspective, linguistic input is converted into propositional symbols, integrated with the knowledge stored in long-term memory, in order to constitute comprehension. In this context, the term “concept” is used to refer to a category of abstract, amodal entities. However, if concepts are entirely represented symbolically, what are the fundamental differences between human language comprehension and artificial intelligence (AI)?

The view of symbolic representation has been challenged by the embodied view. This alternative framework suggests that concepts are fundamentally grounded in bodily action and perception, emphasizing the role of physical experience in shaping mental representations. Language is linked to the real world through the medium of concepts. Although linguistic symbols are arbitrary, concepts are derived from the real world. Embodied theories of cognition hold that thought is grounded in the same neural systems that govern sensation, perception and action (Barsalou, 1999; Glenberg and Kaschak, 2002). Thus, concepts are not typically processed in isolation but rather situated in background settings, events, and introspections. When an individual engages in an action or experiences an emotion in a given context, the brain generates a sensory-motor state that is congruent with the situation. As a situation is repeated, the concepts that process it become increasingly established in memory (Barsalou, 2008, 2023). Therefore, concepts are represented perceptually, rather than symbolically.

An increasing number of behavioral studies have indicated that concepts in first language are grounded in perceptual and experiential information (Stanfield and Zwaan, 2001; Pecher et al., 2009; Glenberg and Gallese, 2012; Marino et al., 2013; Fino et al., 2016). Furthermore, recent studies have indicated that the core language areas in the human neocortex, in conjunction with sensorimotor structures, form a highly interactive system that serves as the foundation for first language comprehension (Fernandino et al., 2013; Davis et al., 2015; Buccino et al., 2017; Vukovic et al., 2017; Gijssels et al., 2018). In the acquisition of their first language, individuals naturally tend to link words to corresponding objects and events within their environment. This recurrent coupling of linguistic symbols with tangible, real-world phenomena plays a pivotal role in the development of perceptually grounded concepts, with some notable exceptions, such as negated events (Vanek et al., 2024). Through this process, the human mind integrates abstract linguistic information with rich sensory experiences, thereby establishing a comprehensive cognitive framework for understanding and interacting with the world.

Four categories of embodiment have been proposed: unembodiment, secondary embodiment, weak embodiment, strong embodiment (Meteyard et al., 2012). In the unembodied view, sensorimotor information plays no role in conceptual representation (Machery, 2006; Meteyard et al., 2012). In the secondary embodiment view, concepts are amodal but linked to sensorimotor information (Patterson et al., 2007; Mahon and Caramazza, 2008). Both the unembodiment and secondary embodiment theories are compatible with the ideas of classical cognitive science, according to which cognition consists of computation over amodal and arbitrary symbols, and perceptual organs serve only as peripheral devices (Meteyard et al., 2012). The weak embodiment view holds that cognition requires some sensorimotor activation. Semantic representations are at least partly constituted by sensory-motor information (Vigliocco et al., 2004). The strong embodiment view holds that cognition cannot occur without sensorimotor activation (Tuena et al., 2023). Despite these advances, the nature of conceptual representation remains ambiguous. Specifically, whether the degree of embodiment varies across the first language and the second language of a multilingual individual is an open question that warrants further investigation. The present study investigates the differences in perceptual representations among multilingual individuals. By exploring these differences, we expect to contribute additional substantiation to the theories of weak and strong embodiment, thereby sharpening the comprehension of cognition’s embodied essence.

However, there are some other researchers who oppose the theories of embodiment. It is notable that not all studies provide evidence in support of embodied cognition. For instance, in certain studies, lesions to the motor cortex did not result in deficits in action word processing (Papeo et al., 2010; Maieron et al., 2013), indicating that activation of sensorimotor structures was not necessarily required in processing language. Moreover, the precise functional role of the sensorimotor activation remains a matter of debate. One hypothesis is that embodied mechanisms are an inseparable and functionally crucial part of language processing (Vukovic et al., 2017). An alternative hypothesis is that embodied mechanisms represent a by-product of language processing, and are therefore functionally redundant and irrelevant to efficient semantic comprehension (Mahon and Caramazza, 2008; Lotto et al., 2009).

There is still no conclusive answer to the question of the nature of conceptual representation. The conceptual representation of the multilinguals are of crucial importance for the advancement of our comprehension of language processes. Second language research is beneficial for the investigation of the developmental factors influencing the formation of embodied mechanisms. However, compared to first language (L1) research, studies on second language (L2) conceptual representations are relatively scarce, yielding somewhat mixed results. Some findings suggest that L2 concepts are perceptually represented as L1 concepts (De Grauwe et al., 2014; Dudschig et al., 2014; van Zuijlen et al., 2024). It is assumed that the perceptual representations in the first language could be equally transferred to the second language processing through the shared concepts. However, other findings show no perceptual representation in second language comprehension (Chen et al., 2020) or weaker embodiment in second language (Vukovic and Shtyrov, 2014; Foroni, 2015; Wang and Zhao, 2024), suggesting that there are quantitative or qualitative differences between L1 and L2 perceptual representations during language comprehension. Further research is required to gain a deeper understanding of the manner in which the diverse linguistic repertoires of multilinguals interact with conceptual representations and the real-world experiences of individuals.

What factors influence the display of perceptual representations in language comprehension? Under the embodiment view, memory works in the service of action and perception. Language proficiency is also an important potential factor affecting perceptual representations. Thus, this study investigates how memory and language proficiency influence the perceptual representation of not only the first language but also the second language and even the third language in multilingual individuals.

Memory plays a significant role in conceptual representation. The majority of contemporary researchers support the view that perception and cognition are inextricably linked. The perceptual system, for instance, is responsible for extracting information from the environment and transmitting it to higher cognitive functioning systems such as memory and thinking. Perceptual representation is an immediate and automatic form of representation in working memory. When relevant information in language comprehension enters long-term memory, it is represented as propositional symbols in the mind (Wang et al., 2005). However, Barsalou (1999) asserts that once a perceptual state is created, some of the perceptual information is extracted through selective attention and stored in long-term memory. Pecher et al. (2009) employed a delayed task to support the notion that native speakers exhibited perceptual representations in the long-term memory stage. Our study expects to examine whether multilinguals also possess perceptual representations in their long-term memory.

The present study used the sentence-picture verification paradigm (Pecher et al., 2009) to investigate perceptual representations in multilingual participants. This paradigm is a typical approach to investigating the activation of fine-grained perceptual features during language comprehension. There are two versions of this paradigm: an immediate version and a delayed version. In the immediate sentence-picture verification paradigm, participants listen to a sentence and then immediately see a picture, after which they decide whether the object in the picture is mentioned in the preceding sentence. The delayed sentence-picture verification paradigm comprises two phases: a sentence judgment phase and a picture recognition phase. In the sentence judgment phase, participants are presented with sentences and required to decide whether the sentences are meaningful or not. Subsequently, in the picture recognition phase, participants are instructed to look at pictures and decide whether the objects in the pictures were mentioned in the preceding sentences. The key feature of the sentence-picture verification paradigm is that the perceptual features of the objects mentioned in sentences can either match or mismatch those of the objects depicted in the pictures. To illustrate, the implied shape of an eagle in a sentence (e.g., “There was an eagle in the sky”) can match or mismatch the shape of an eagle in a picture (e.g., an eagle with wings stretched out or with wings drawn in). It is critically important to note that the target perceptual feature is task-irrelevant and only implied in the sentences.

The rationale of the paradigm is as follows: If concepts are represented perceptually, participants will engage in perceptual simulations, and demonstrate sensitivity to the implied features. In other words, they will be faster to verify pictures that match the perceptual features than those that do not. Typically, the reaction time is facilitated when the implied features match the visually presented targets (i.e., the match effect, as observed by Stanfield and Zwaan, 2001). We anticipated that perceptual representations would be stronger in the immediate task associated with working memory than in the delayed task linked to long-term memory.

Language proficiency can be another important factor affecting conceptual representations. Current studies mainly contrast the first language (L1) with the second language (L2). Yet, in addition to differences in proficiency, the divergent acquisition processes that define first and second languages stand as a fundamental distinction in their characterization. In an effort to examine the effects of proficiency, we involved a high-proficiency second language and a low-proficiency third language (L3) in our study. We hypothesized that perceptual representations would more robust and readily accessible in L1 due to their strong embodiment and lifelong exposure. High-proficiency L2 can embody perceptual representations similarly to L1, given extensive practice and usage. Low-proficiency L3 may have weaker perceptual representations due to less frequent use and less embodied experience.

## 2 Experiment 1

### 2.1 Research question

Previous studies have demonstrated the existence of perceptual representation in L1 sentence comprehension (Zwaan et al., 2002; Pecher et al., 2009). Nevertheless, no perceptual representation has been observed in L2 sentence comprehension in tasks involving long-term memory (Chen et al., 2020). Therefore, an immediate sentence-picture verification task was employed to replicate the previous studies. The aim of Experiment 1 was to investigate whether the high-proficiency L2 demonstrated perceptual representations, as well as the L1, and whether the low-proficiency L3 could demonstrate perceptual representation in some way during the working memory stage.

### 2.2 Participants

In the field of linguistics, the terms “first language,” “mother tongue,” and “native language” are used to describe the language a person acquires from birth or early childhood. This is the language they acquire naturally and effortlessly as part of their primary socialization process. The first language exerts a profound influence on an individual’s perception of the world and cognitive development. It serves as the foundation for learning additional languages (second language, L2, or third language, L3, etc.).

At the time of recruitment, the participants in Experiment 1 were born, raised, and continued to reside and pursue studies in the Pearl River Delta where Cantonese was the dominant dialect. In this experiment, Cantonese was considered as the first language (L1) with a daily-based acquisition. Previous studies have highlighted the difficulty in determining the language proficiency of L2. The participants in Experiment 1 initiated their formal education in Mandarin as the official language at approximately 7 years of age, when they began primary school. As Chinese college students, our participants have been speaking and using Mandarin in formal settings for more than 10 years. This indicated a very high level of proficiency in Mandarin. Consequently, Mandarin was considered to be the high-proficiency L2 in the present experiment. Furthermore, our participants primarily learned and used English as a foreign language in educational settings and other occasional contexts. The participants were not majoring in English. At the time of recruitment, they had not yet passed the College English Test-Band 6, indicating a relatively lower level of English proficiency. English was considered to be the low proficiency L3 in the present experiment.

A total of 36 students from South China Normal University in Guangzhou China were recruited to participate in the experiment, including 23 female students and 13 male students. The mean age was 21.41 years (SD = 1.048). A survey of language background was conducted to identify native Cantonese speakers. At the time of testing, the participants spoke Mandarin as an official language and English as a foreign language. They were non-English majors, and had passed the College English Test-Band 4, but had not yet passed the College English Test-Band 6. All participants confirmed to be right-handed and reported no history of hearing impairment, reading difficulties, or imaging-related disabilities. They were compensated with 35 RMB for their participation in the experiment.

### 2.3 Materials

A total of 60 experimental sentence-picture sets were created (see Table 1). Each experimental set consisted of six sentences and two black and white pictures. The six sentences described an object with two different implied shapes in three language versions (Cantonese, Mandarin, and English). The two pictures depicted two different shapes of the same object. In sum, 360 sentences and 120 pictures were used as experimental materials.

**Table 1:** Example for materials in each experimental set.

Languages	Audio sentences	Pictures
L1: Cantonese	个度飞住只鹰	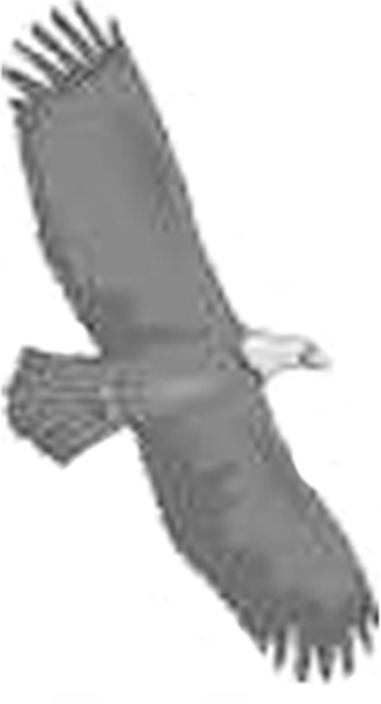
	鹰窦入边有只鹰	
L2:Mandarin	天空中有只老鹰	
	巢穴中有只老鹰	
L3:English	There was an eagle in the sky	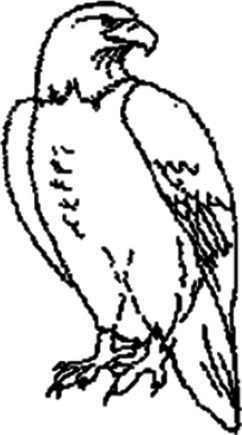
	There was an eagle in the nest	

Cantonese has no written form. The transliterated sentences in the table are just for illustration. All the sentence materials are in audio version.

Additionally, there were 60 filler sentences (20 sentences X 3 language versions) and 60 filler pictures. For instance, the sentence “There was a panda in the zoo” was a filler sentence. A picture of “a panda” was a mentioned filler picture, whereas a picture of “a camel” was an unmentioned filler picture.

Both experimental and filler sentences were recorded by native Cantonese, native Mandarin and native English speakers, respectively. The materials were evaluated for comprehensibility and imageability by 15 students who did not participate in the formal experiment. The materials were rated using a Likert-5-point scale, ranging from 1 (Not at all comprehensible/Not at all imaginable) to 5 (Extremely comprehensible/Extremely imaginable). For comprehensibility, the mean score across all materials was 4.89, indicating a high level of clarity and understandability. Similarly, for imageability, the materials received a mean score of 4.14, reflecting a strong capacity for evoking mental images. The results showed that the materials were valid. The experimental and filler materials used in Experiment 1 can be accessed via https://osf.io/m78tq/.

### 2.4 Procedure

Experiment 1 was a 3 (Cantonese/Mandarin/English) × 2 (match/mismatch) design. The dependent variables were reaction time and hit rate of picture recognition.

The entire experiment was displayed on a computer screen using E-prime. The experiment consisted of three language blocks, each containing 40 trials (10 match trials, 10 mismatch trials, and 20 filler trials). The trials within each block were presented in a random order. The three language blocks were counterbalanced. Moreover, eight additional sentences and eight pictures (4 related and 4 unrelated) were used in the practice trials.

**Figure 1:**
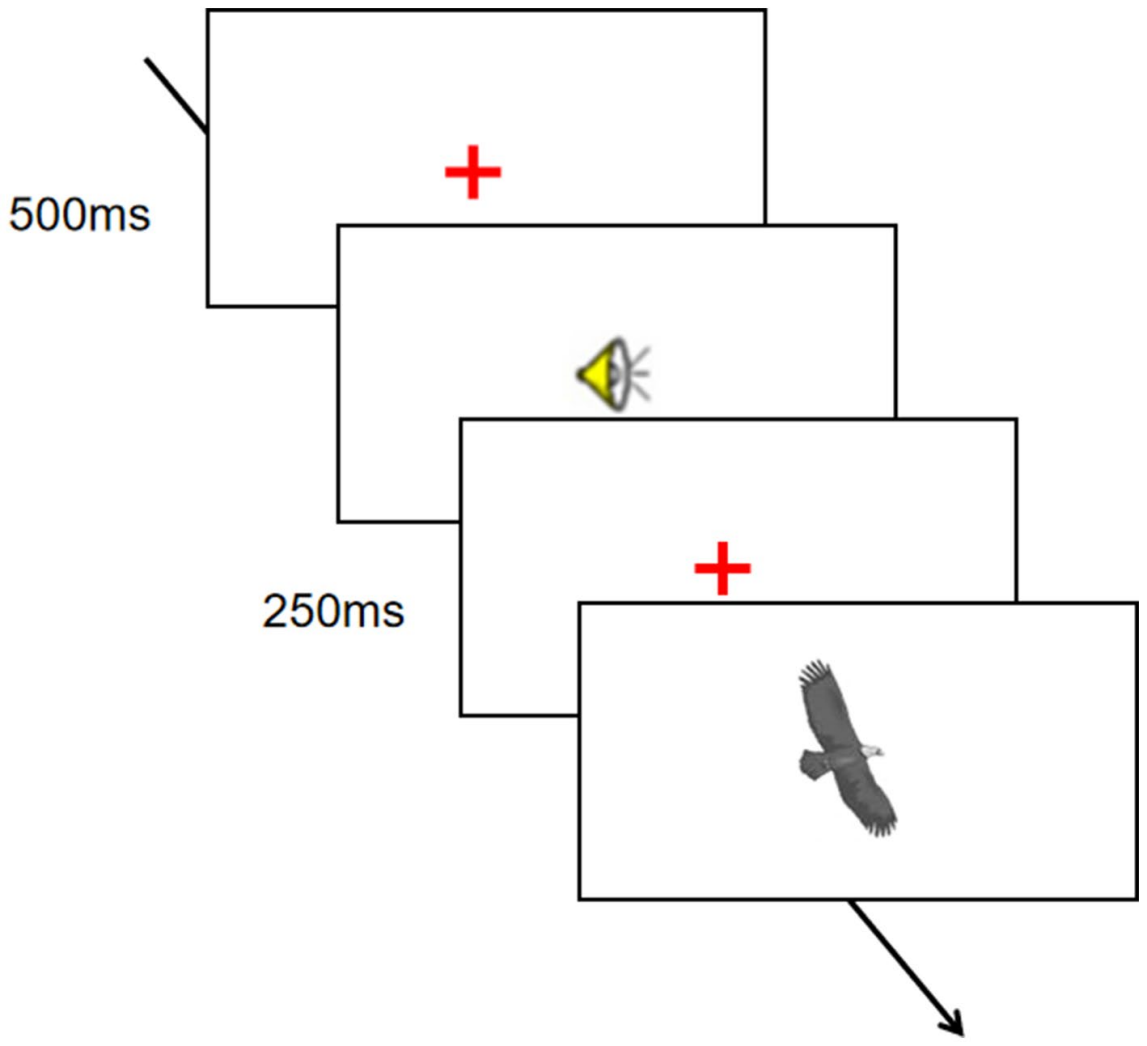
The immediate sentence-picture verification task in Experiment 1.

In each trial (see Figure 1) of the immediate sentence-picture verification paradigm, participants were first presented with a fixation (a red cross) in the center of the screen for a duration of 500 ms. They either maintained gaze on the fixation point or pressed the Space key to proceed to the next step. Subsequently, the speaker icon was displayed in the center of the screen, while the headset played the sentence. Participants were required to listen to the sentence carefully. After the sentence, a fixation point was displayed in the center of the screen for a duration of 250 ms. Participants were also given the option of pressing the Space key to proceed. Subsequently, a picture was displayed in the center of the screen. Participants were required to determine whether the picture was mentioned in the sentence that had just been heard. Participants pressed the “J” key for a picture that was mentioned or the “F” key for a picture that was not mentioned. The reaction time and hit rate of the participants were automatically recorded by the computer. The whole experiment took about 15 min.

### 2.5 Results and discussion

Fillers and practice items were removed from the data set. Two participants were excluded from the analysis as they failed to comply with the experimental instructions, consistently pressing the same key throughout the task. For the remaining participants, mean hit rates for picture recognition were calculated. The data of three participants were deleted due to their mean hit rate being below 60%. The final data set consisted of 1,860 observations for 31 participants. Reaction times for incorrect responses (8%) were excluded. For each condition 3 (Cantonese/Mandarin/English) × 2 (match/mismatch), trials slower than the mean reaction time plus 3 × standard deviation (1.5%) were also excluded from the analysis. After data cleaning, there were 1,683 observations left for reaction times. The specific distribution was as follows: 569 observations remained in Cantonese, 584 in Mandarin, and 530 in English. The hit rates and reaction times were then analyzed.

The mean hit rates and reaction times for the different conditions in the picture recognition task are presented in Tables 2, 3. As illustrated in Tables 2, 3, in the match condition, Cantonese exhibited the fastest response time (630.33 ms ± 144.30), followed by Mandarin (669.05 ms ± 197.08), and English exhibited the slowest response time (890.66 ms ± 261.79). The hit rates of Cantonese and Mandarin were comparable (0.94 ± 0.08, 0.97 ± 0.05), while the hit rate of English was the lowest (0.87 ± 0.10). The same was true in the case of the mismatch condition. This finding indicates that participants did not prioritize speed or accuracy over the other in the context of our experimental design. The analysis showed no trade-off between speed and accuracy.

**Table 2:** Mean hit rates of picture recognition in Experiment 1.

Language	Match type	Mean hit rate	SD	Sig (*p* < 0.05)
L1: Cantonese	Match	0.94	0.08	*t* = 0.862
	Mismatch	0.92	0.09	*p* = 0.395
L2: Mandarin	Match	0.97	0.05	*t* = 2.352
	Mismatch	0.93	0.96	*p* = 0.025^*^
L3: English	Match	0.87	0.10	*t* = −0.538
	Mismatch	0.89	0.13	*p* = 0.595

**Table 3:** Mean reaction times of picture recognition in Experiment 1 (ms).

Language	Match type	Mean RT	SD	Sig (*p* < 0.05)
L1: Cantonese	Match	630.33	144.30	*t* = −2.695
	Mismatch	669.59	157.96	*p* = 0.011^*^
L2: Mandarin	Match	669.05	197.08	*t* = −1.043
	Mismatch	690.44	165.49	*p* = 0.305
L3: English	Match	890.66	261.79	*t* = −1.281
	Mismatch	925.16	278.85	*p* = 0.210

Table 2 provides a summary of the mean hit rates that were observed across the six conditions in the picture recognition study. A 3 (Cantonese/Mandarin/English) × 2 (match/mismatch) repeated measures ANOVA was performed on the hit rates. There was a significant effect of language, *F* (2,60) =7.019, *p* = 0.013. There was no significant effect of match type, *F* (1,30) = 1.849, *p* = 0.184. There was no interaction effect between language and match type, *F* (2,60) = 1.559, *p* = 0.221. The results of the RANOVA analysis indicated a significant effect of language. This was primarily due to the lower hit rate of L3 compared to L1 and L2. This was attributed to the fact that L3 is a low-proficient foreign language. Upon closer examination of the specific conditions, the hit rate for the match condition was 0.94 for L1 and 0.97 for L2; the hit rate for the mismatch condition was 0.92 for L1 and 0.93 for L2. Statistical analysis did not reveal any significant differences between the match conditions for L1 and L2 (*t* = −1.489, *p* = 0.147 > 0.05), nor between the mismatch conditions for L1 and L2 (*t* = −0.130, *p* = 0.897 > 0.05). L1 serves as the primary language used in daily communicative practices, and L2 has been adopted with high proficiency levels, functioning as the official language. The hit rates of L1 and L2 were both very high, making it difficult to show significant differences. The results of the RANOVA analysis indicated a significant effect of language. This was primarily due to the lower hit rate of L3 compared to L1 and L2. This was attributed to the fact that L3 is a low-proficient foreign language.

Of greater interest was the comparison between the match and mismatch conditions for each language. The analysis of the RANOVA data revealed no significant difference between the match types and no significant interaction. Consequently, further analysis of the hit rate was not conducted, and greater emphasis was placed on the reaction time than on the hit rate.

**Figure 2:**
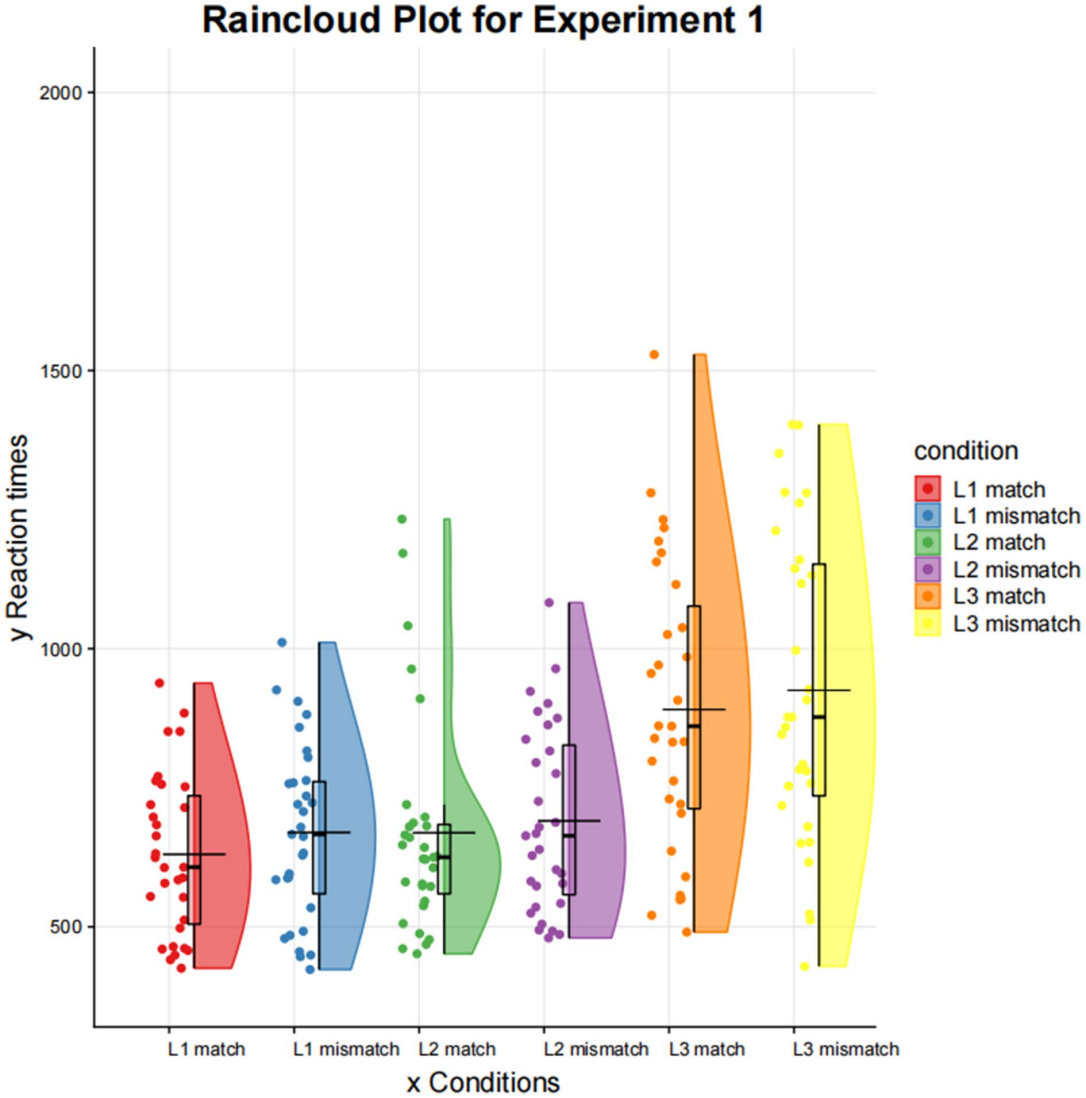
Reaction time of picture recognition in Experiment 1.

The mean reaction times for the different conditions in the picture recognition task are shown in Table 3 and Figure 2. A 3 (Cantonese/Mandarin/English) × 2 (match/mismatch) repeated measures ANOVA was performed on the mean reaction times. A significant effect was observed for language, *F* (2,60) =30.887, *p* = 0.000. More importantly, a significant effect was observed for match type, *F* (1,30) =6.030, *p* = 0.020. There was no interaction effect between language and match type, *F* (2,60) =0.20, *p* = 0.658.

Given that the potential interaction effect was not statistically significant, we did not proceed with simple effects analysis. The hypothesis under consideration pertained to the differences between match and mismatch responses under varying language conditions. Therefore, paired samples *t*-test was employed to further investigate the discrepancies under varying conditions. The paired *t*-test permitted the direct testing of the hypothesis of mean differences between match and mismatch conditions within each language. This hypothesis remains informative even in the absence of an interaction effect at the overall level.

A paired samples *t*-test analysis revealed that the reaction time of the match condition was significantly shorter than that of the mismatch condition for Cantonese comprehension (*t* = −2.695, *p* = 0.011). The results demonstrated that there was no significant match effect for Mandarin (*t* = −1.043, *p* = 0.305) and English (*t* = −1.281, *p* = 0.210) comprehension. Thus, Experiment 1 showed that the match effect was significant within L1, but not within L2 or L3.

The aim of Experiment 1 was to examine the perceptual representations generated in working memory by different languages of multilinguals. The results indicated that match effect was observed only in L1, suggesting that perceptual representations were generated in the comprehension of the first language. In contrast, the later-learned languages (L2 and L3) did not exhibit a match effect, suggesting that mismatching perceptual representations were integrated comparable ways to matching perceptual representations in these languages. In other words, the findings suggested that the multilinguals generated perceptual representations only in their L1 comprehension, but not in the high-proficiency L2, even during the working memory stage, let alone low-proficiency L3. The comparison between L2 and L3 showed no significant effect of language proficiency on perceptual representations. This outcome partially contradicted our initial hypothesis. It is reasonable to find no match effect in L3 comprehension. The third language was characterized by lower proficiency level, slower activation and less depth in processing, which likely hindered the manifestation of perceptual representations. Surprisingly, the high-proficiency second language also failed to demonstrate perceptual representations. This outcome suggests that high language proficiency does not necessarily evoke perceptual representations.

## 3 Experiment 2

### 3.1 Research question

The participants in Experiment 1 were Cantonese-speaking college students living and studying in the Cantonese-speaking region. If we were to change our participants to Mandarin-speaking college students living and studying in the Pearl River Delta, and use a delayed sentence-picture verification paradigm, would the L1 match effect still be found in the long-term memory stage?

In Experiment 2, a delayed sentence-picture verification paradigm was employed to investigate the differences in perceptual representations between the first language (L1: Mandarin) and a foreign language (L2: English) at the long-term memory stage.

### 3.2 Participants

A total of 24 students from South China Normal University in Guangzhou China were recruited to participate in the Experiment 2, including 20 female students and 4 male students. The mean age of the participants was 21.25 years (SD = 0.897). A survey of language background was conducted to identify students from northern China studying at SCNU. At the time of recruitment, they had been speaking Mandarin as their mother tongue since childhood and they had learned English as a foreign language for about 8 years. They were not English majors, but had passed the College English Test-Band 6, indicating that they had achieved a satisfactory level of foreign language proficiency. All participants confirmed to be right-handed and reported no history of hearing impairment, reading difficulties, or imaging-related disabilities. They were compensated with 35 RMB for their participation in the experiment.

### 3.3 Materials

The target sentences (Mandarin and English sentences) and target pictures in Experiment 2 were the same as those in Experiment 1. We added filler sentences and pictures to the materials for the delayed sentence-picture verification task. For instance, the sentence “There was a panda in the zoo” was a meaningful filler sentence, whereas the sentence “There was a store in the student” was an meaningless filler sentence. A picture of “a panda” was a mentioned filler picture, whereas a picture of “a camel” was an unmentioned filler picture. In total, there were 160 meaningful target sentences (2 languages × 2 shapes × 40 sets), 80 target pictures (2 shapes × 40 sets), 80 filler sentences (20 meaningful sentences and 60 meaningless sentences), and 80 filler pictures. The experimental and filler materials used in Experiment 2 can be accessed via https://osf.io/m78tq/.

### 3.4 Procedure

Experiment 2 was a 2 (Mandarin/English) × 2 (match/mismatch) design. The dependent variables were reaction time and hit rate of picture recognition.

**Figure 3:**
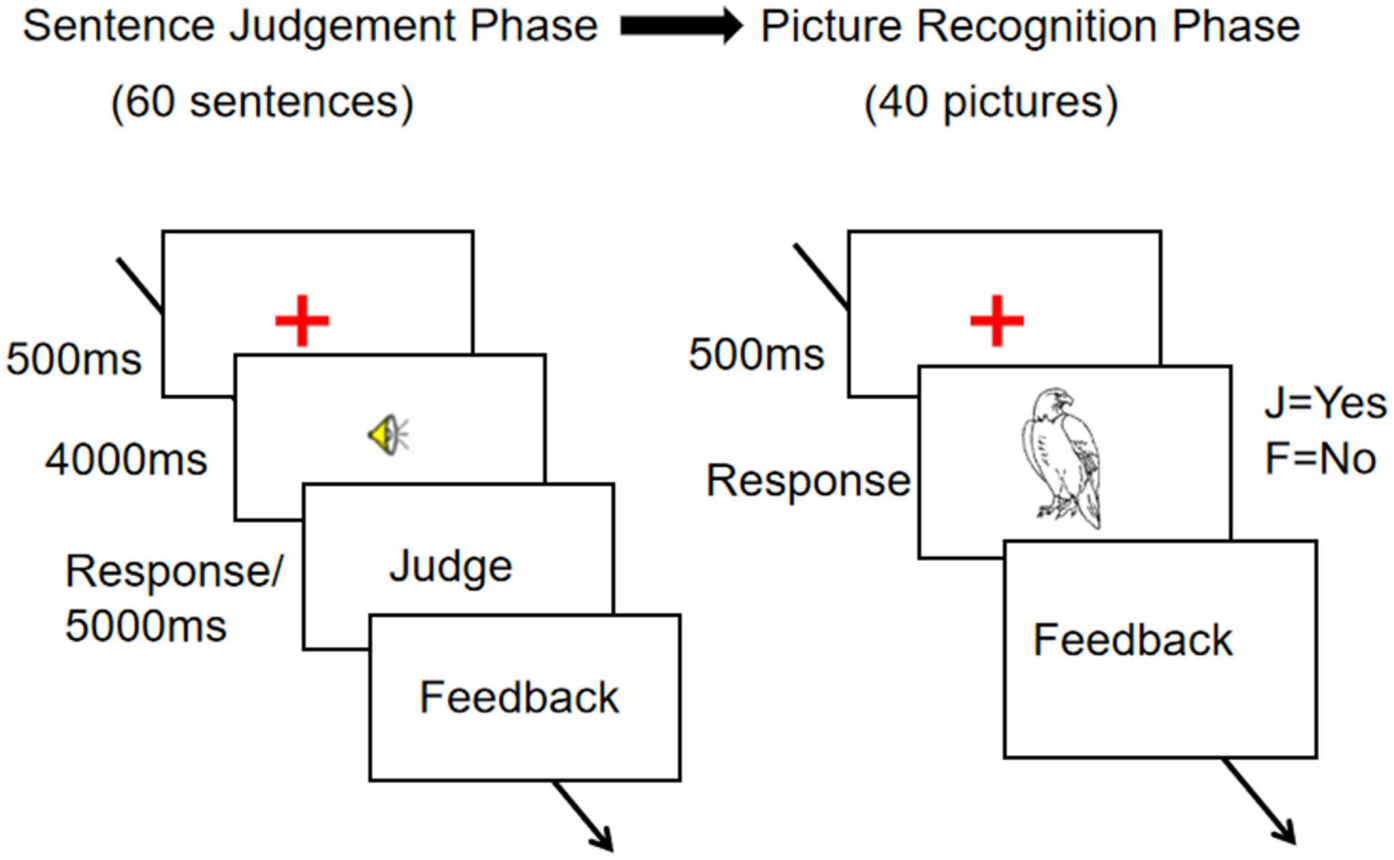
The delayed sentence-picture verification task in Experiment 2.

The entire experiment was displayed on a computer screen using E-prime (see Figure 3). The delayed sentence-picture verification paradigm (Pecher et al., 2009; Chen et al., 2020) in this study consisted of two phases: the sentence judgment phase and the picture recognition phase. There were two language blocks (Mandarin and English).

In each trial of the sentence judgment phase, participants were first presented with a fixation (a red cross) in the center of the screen for a duration of 500 ms. They were then instructed to either maintain gaze on the fixation point or press the Space key to proceed to the next step. Subsequently, the speaker icon was displayed in the center of the screen, while the headset played the sentence, with a duration of 4,000 ms. After that, participants were required to decide whether the sentence was meaningful or not. A meaningful sentence required a “yes” response (press “J” on the keyboard) and a meaningless sentence required a “no” response (press “F” on the keyboard). In the event that the participant failed to respond within 5,000 milliseconds, the subsequent step would be initiated automatically. Feedback was provided after each response. In each language block, the participants were presented with a series of 60 sentences, after which they were required to complete a picture recognition task involving 40 pictures.

In each trial of the picture recognition phase, participants were first presented with a fixation (a red cross) in the center of the screen for a duration of 500 ms. They were then required to either maintain gaze on the fixation point or press the Space key to proceed to the next step. Subsequently, a picture was displayed in the center of the screen. They were required to decide whether the picture was mentioned in the sentences they had heard in the previous phase. A mentioned picture required a “yes” response (press “J” on the keyboard) and an unmentioned picture required a “no” response (press “F” on the keyboard). Feedback was provided after each response. Reaction times and hit rates of the participants were automatically recorded by the computer.

All trials in each block were presented in a random order. Two language blocks were counterbalanced across participants. In addition, 8 sentences (4 meaningful and 4 meaningless) and 8 pictures (4 related and 4 unrelated) were used in practice trials. The whole experiment took about 20 min.

### 3.5 Results and discussion

Fillers and practice items were removed from the data. One invalid participant was excluded because she did not complete the whole process. For the remaining participants, mean hit rates for picture recognition were calculated. Two participants’ data were deleted because their mean hit rate was less than 60%. The final data set consisted of 840 observations for 21 participants. The reaction times of the incorrect responses (26%) were excluded. For each condition, trials slower than the mean reaction time plus 3 × standard deviation (1.25%) were also excluded from the analysis. After data cleaning, there were 612 observations left for reaction times. The specific distribution was as follows: 309 observations remained in Mandarin, and 303 in English. Hit rates and reaction times were then analyzed.

The mean hit rates and reaction times for the different conditions in the picture recognition task are presented in Tables 4, 5. As illustrated in Tables 4, 5, in the match condition, Mandarin exhibited the faster response time (973.57 ms ± 182.68) and English exhibited the slower response time (1107.09 ms ± 289.35). Mandarin exhibited a higher hit rate (0.77 ± 0.18) and English a lower hit rate (0.74 ± 0.14). The same was true in the case of the mismatch condition. This finding indicated that participants did not prioritize speed or accuracy over the other in the context of our experimental design. The analysis showed no trade-off between speed and accuracy.

**Table 4:** Mean hit rates of picture recognition in Experiment 2.

Language	Match type	Mean hit rate	SD	Sig (*p* < 0.05)
L1: Mandarin	Match	0.77	0.18	*t* = 1.027
	Mismatch	0.73	0.13	*p* = 0.317
L2: English	Match	0.74	0.14	*t* = 0.113
	Mismatch	0.73	0.18	*p* = 0.911

**Table 5:** Mean reaction times of picture recognition in Experiment 2 (ms).

Language	Match type	Mean RT	SD	Sig (*p* < 0.05)
L1: Mandarin	Match	973.57	182.68	*t* = −2.975
	Mismatch	1061.66	226.01	*p* = 0.007^**^
L2: English	Match	1107.09	289.35	*t* = 0.718
	Mismatch	1073.23	321.10	*p* = 0.481

The mean hit rates for the 4 conditions in the picture recognition task are shown in Table 4. A 2 (Mandarin/English) × 2 (match/mismatch) repeated measures ANOVA was performed on the picture recognition hit rates. There was no significant effect of language, *F* (1,20) =0.168 *p* = 0.686. There was no significant effect between match types, *F* (1,20) = 0.464, *p* = 0.504. There was no interaction effect between language and match type, *F* (1,20) = 0.670, *p* = 0.423. Therefore, further analysis of the hit rate was not conducted. A more compelling inquiry would be to compare the match and mismatch conditions for each language. The focus was placed on reaction time rather than on the hit rate.

**Figure 4:**
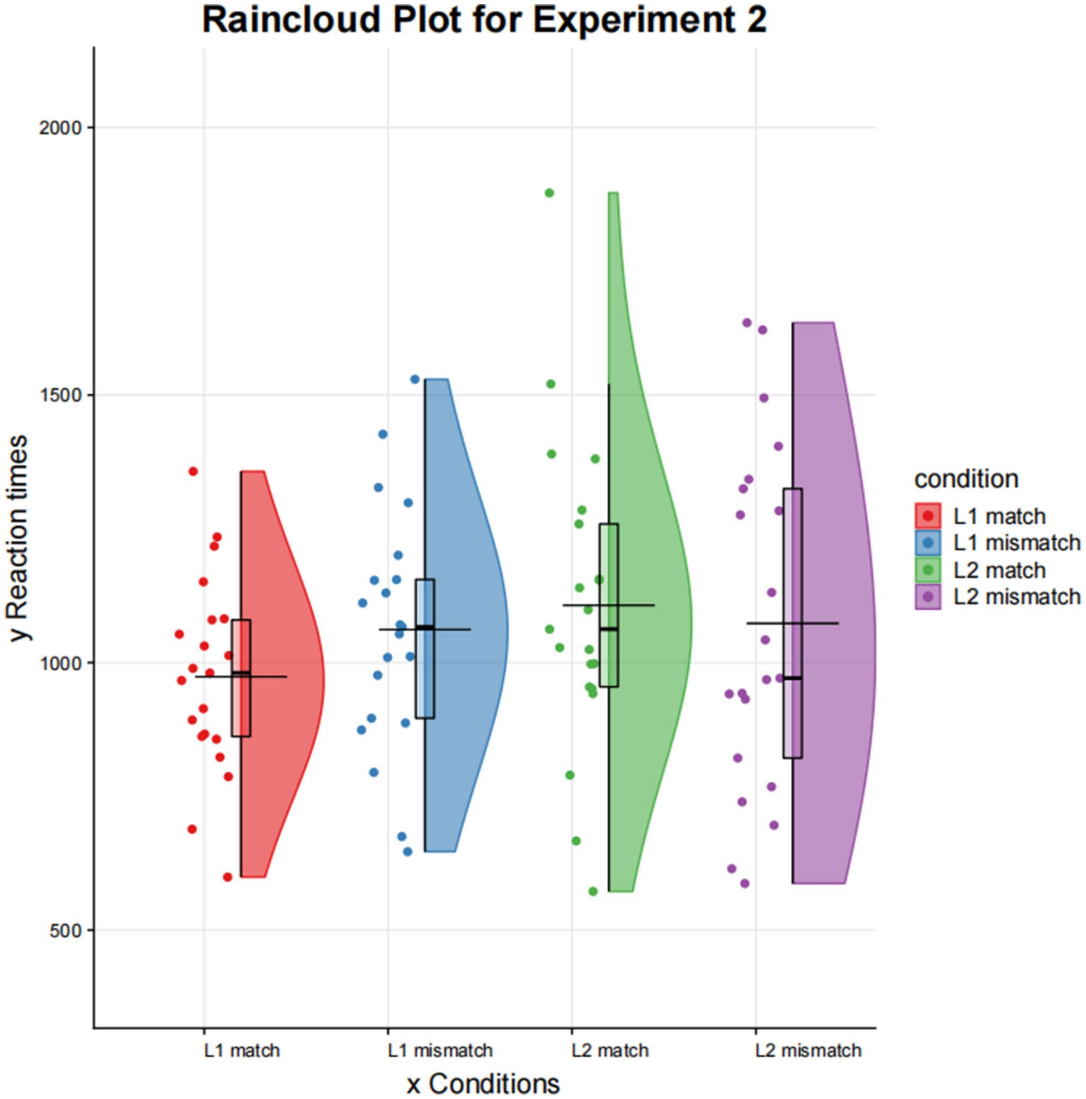
Reaction time of picture recognition in Experiment 2.

The mean reaction times for the different conditions in the picture recognition task are shown in Table 5 and Figure 4. A 2 (Mandarin/English) × 2 (match/mismatch) repeated measures ANOVA was performed on the mean reaction times. There was no significant effect of language, *F* (1,20) =2.139, *p* = 0.159. There was no significant effect between match types, *F* (1,20) =2.570, *p* = 0.125. There was no interaction effect between language and match type, *F* (1,0) =2.939, *p* = 0.102. Overall, the results showed no significant effects in delayed sentence-picture verification. In comparison to the significant effect found in Experiment 1, the findings of Experiment 2 suggest that long-term memory exerts a significant influence on the display of perceptual representation of concepts.

Given that the potential interaction effect was not statistically significant, we did not proceed with simple effects analysis. The hypothesis under consideration pertained to the differences between match and mismatch responses under varying language conditions. Therefore, paired samples *t*-test was employed to further investigate the discrepancies under varying conditions. The paired *t*-test permitted the direct testing of the hypothesis of mean differences between “match” and “mismatch” conditions within each language. This hypothesis remains informative even in the absence of an interaction effect at the overall level.

Paired samples *t*-test analysis showed that the reaction time of the match condition was significantly shorter than that of the mismatch condition in Mandarin comprehension (*t* = −2.975, *p* = 0.007). There was no match effect for English comprehension (*t* = 0.718, *p* = 0.481). The results of Experiment 2 again suggest that the match effect was significant within L1, but not within L2.

In Experiment 2, we switched to a different first language (Mandarin) and employed a delayed sentence-picture verification task. Once more, the match effect was observed in the first language comprehension, but not in the later-learned language (English). This suggests that L1 comprehension was strongly embodied in perceptual experiences, whereas L2 comprehension was unlikely to be. Compared to the immediate sentence-picture verification task, the difference of conceptual presentations between languages was less significant in the delayed sentence-picture verification task. The findings of Experiment 2 suggest that long-term memory plays a role in influencing the display of perceptual representations in first language comprehension.

## 4 General discussion

The aim of the present study was to investigate how different languages were represented by multilinguals. In Experiment 1, we used the immediate sentence-picture verification paradigm to examine perceptual representations in the working memory stage. We found a significant difference in languages. Specifically, we found a match effect within the first language (Cantonese), but no match effect within the second language (Mandarin) or the third language (English). Prior to the study, we hypothesized that both L1 and high proficient L2 would be perceptually represented, with L1 exhibiting stronger embodiment than L2, and low proficient L3 exhibiting weak or even no embodiment. The results yielded partial support for these predictions. The first language was perceptually represented. However, despite the second language being widely spoken as an official language with high proficiency, and the third language being an occasionally used foreign language with lower proficiency, no match effect was observed in their comprehension. This suggests that language proficiency does not have a significant effect on the exhibition of perceptual representations.

In Experiment 2, we employed the delayed sentence-picture verification paradigm to investigate perceptual representations stored in long-term memory. Our results revealed no significant difference between languages at an overall level. Nonetheless, we observed a match effect within the first language (Mandarin), a finding that was absent in the second language (English). These findings suggest that a match effect was consistently present in L1 comprehension, irrespective of whether processing occurs at the working memory stage or the long-term memory stage. Furthermore, this effect held true whether the L1 was Cantonese or Mandarin. The results in our study supports the notion that conceptual representations in the L1 are inherently and persistently perceptual. Given the recurrence of these findings in L1 comprehension, which aligns with previous research (Tomasino et al., 2007; Pecher et al., 2009; Repetto et al., 2013; Nijhof and Willems, 2015; Zwaan, 2016), it is plausible to infer that concepts in the L1 are subconsciously reactivated through perceptual simulation during language comprehension.

Our findings lend support to the notion that first language comprehension is strongly embodied. According to the strong embodiment perspective, sensorimotor activation is a prerequisite for cognitive activities (Tuena et al., 2023). That is to say, when there is cognitive activity, there is sensorimotor activation. Our results from the sentence-picture verification paradigm, where the target perceptual feature was merely implied and task-irrelevant, revealed a match effect in first language processing, even under delayed task conditions. This indicates that perceptual experience is unconsciously engaged and plays an indispensable role in cognitive tasks.

It is more probable that the representation of human language is not exclusively symbolic, but is derived from the human experience of interacting with the world and others. The acquisition of first language through prolonged and varied engagement in authentic social scenarios, lays the groundwork for conceptual development. First language acquisition and cognitive development are intertwined and cannot be completely separated. Therefore, first language implies a stronger sense of emotion and morality than a second language. For instance, individuals may find it relatively effortless to express sentiments such as “I love you” in a foreign language, yet exercise considerable caution when conveying the same message in their native tongue due to the heightened emotional intensity inherent in the latter. This phenomenon is supported by a number of empirical studies (Foroni, 2015; Sheikh and Titone, 2015; Zhao et al., 2020), which indicate that the embodiment of emotion words in first language is stronger than that of emotion words in second language (but for counterevidence see, e.g., Vanek and Tovalovich, 2022).

Moreover, our findings revealed that the match effect in L1 was notably more pronounced in the immediate task compared to the delayed task. The manifestation of these perceptual representations appears to be modulated by memory dynamics. A notable limitation of our study pertains to the absence of task-independent memory assessments. We recognize that failing to incorporate a distinct measure of memory capacity into our experimental design might have introduced variability in participant performance. We recommend that future investigations in this domain should strive to include explicit tests of memory capacity as a control variable. Such enhancements to experimental designs will facilitate a more comprehensive analysis of the data and permit a finer-grained interpretation of the findings.

The situation becomes more complicated with regard to the perceptual representation in later-learned language comprehension. In this study, no match effect was found in L2 or L3, whether of high or low proficiency, whether in working memory stage or long-term memory stage. Our findings are consistent with those of some previous studies (Vukovic and Shtyrov, 2014; Foroni, 2015; Sheikh and Titone, 2015; Chen et al., 2020), providing further support for the view that conceptual representations in L2 are different from those in L1. For instance, Foroni’s (2015) study showed when participants read affirmative sentences, the magnitude of somatic activation was smaller in L2 than in L1; unlike in L1, there was no relaxation of the relevant muscles when participants read negative sentences in L2. Foroni (2015) concluded that embodiment was only partial in L2. Also, Vukovic and Shtyrov’s (2014) study found important quantitative differences between L1 and L2 sensorimotor brain activity in language comprehension. Therefore, it is reasonable to consider that concepts in first language are represented perceptually, whereas concepts in languages learned later are represented in a different way, probably mainly represented by propositional symbols.

Indeed, there have been other previous studies that have provided support for the notion of L2 embodiment. Previous studies have primarily focused on specific word types during the working memory phase, such as emotion words (Dudschig et al., 2014), cognate words (De Grauwe et al., 2014), action words (García-Gámez and Macizo, 2018), and spatial words (Ahlberg et al., 2017). It can be posited that these specific word types may be more likely to elicit embodied responses in immediate situations. These findings lend support to the hypothesis that second and third languages, acquired later in life, exhibit weak embodiment. The weak embodiment view posits that cognitive processes may benefit from sensorimotor activation, though such activation is not considered an essential component for the execution of cognitive tasks (Meteyard et al., 2012). It is possible that L2 comprehension may result in the generation of a certain degree of perceptual representation under certain conditions. Future research could concentrate on the circumstances under which L2 comprehension leads to the generation of perceptual representations.

Language proficiency can be one of the factors influencing the manifestation of perceptual representations. In our study, no match effect was observed in highly proficient L2 or L3 with low proficiency, even in the immediate task. This observation suggests that cognitive tasks can be effectively carried out without the reliance on embodied representations, providing empirical evidence for the weak embodiment in second and third language comprehension. The proficiency level of a language, whether it is the high-proficiency L2 or low-proficiency L3, does not appear to be a decisive factor in the manifestation of perceptual representation. Instead, the mode of language acquisition, whether it is the L1 or the languages learned later (L2 and L3), seems to exert a more significant influence. This suggests that the early, immersive, and experiential learning process characteristic of L1 acquisition may lead to deeper and more integrated perceptual representations than those learned later in life under more deliberate and analytical learning conditions. However, the participants in our studies were late multilinguals. L2 and L3 were learned explicitly in a school setting. Future research could explore whether early bilinguals have perceptual representations in two parallel languages, given that both languages are learned in real-life contexts and synchronized with cognitive development. It can be hypothesized that there is an overlap of embodied information between the two different languages.

Taken together, we speculate that multilinguals’ conceptual representations should not be viewed as an all-or-nothing mechanism; instead, they operate along a continuum where the degree of sensory-motor involvement can vary widely. Conceptual representations are both perceptual and symbolic.

On the one hand, the perceptual representation is arguably the most significant feature of human language. Human language is rich in emotion, cultural background and individual experience. Metaphors, rhetoric, and other devices are employed to convey complex emotions and subtle meanings. It is reasonable to posit that human language, particularly first language, must be perceptually representational, rather than merely propositional symbolic.

On the other hand, symbolic representation is also essential to human cognition and language. The symbolic nature of concepts is crucial to the productivity and flexibility of language and thought. More specifically, L1 concepts are likely to be mainly represented perceptually, whereas L2 concepts are usually characterized by symbolic representations. The acquisition of L1 has already established a cognitive framework for L2 comprehension. In order to reduce the cognitive load and facilitate the processing of linguistic information, L2 comprehension does not require the simulation of perceptual experiences in the mind. Instead, L2 comprehension can be linked to the semantic network of the L1. In general, L2 concepts are not directly derived from sensorimotor experiences. L2 embodiment is transferred from L1 through shared concepts. Sensorimotor activation in L1 and non-L1 languages should be different, with weaker connections to the sensorimotor cortex for the non-L1 (Dudschig et al., 2014). The manifestation of perceptual representations in later-learned L2 and L3 is subject to certain constraints.

In conclusion, our study suggests that concepts in first language are mainly represented perceptually because first language and cognition develop simultaneously from interactive human life. Concepts in second and third language are mainly represented symbolically, reflecting the cognitive economy of the brain. Moreover, language acquisition style is found to be a crucial factor impacting perceptual representation, while language proficiency level may not necessarily play a significant role. The manifestation of these perceptual representations is also subject to memory processes, with stronger perceptual representations in first language in working memory relative to long-term memory.

## Data availability statement

The raw data supporting the conclusions of this article will be made available by the authors, without undue reservation.

## Ethics statement

The studies involving humans were approved by School of Psychology, South China Normal University. The studies were conducted in accordance with the local legislation and institutional requirements. The participants provided their written informed consent to participate in this study.

## Author contributions

DC: Conceptualization, Methodology, Writing – original draft, Writing – review & editing. JS: Investigation, Methodology, Writing – original draft. RW: Supervision, Writing – review & editing.
